# 6-(3,5-Dimethyl­benz­yl)-5-ethyl-1-[(3-phenyl­prop­oxy)meth­yl]-1,2,3,4-tetra­hydro­pyrimidine-2,4-dione

**DOI:** 10.1107/S1600536811055723

**Published:** 2012-01-11

**Authors:** Nasser R. El-Brollosy, Ali A. El-Emam, Omar A. Al-Deeb, Seik Weng Ng

**Affiliations:** a Department of Pharmaceutical Chemistry, College of Pharmacy, King Saud University, Riyadh 11451, Saudi Arabia; b Department of Chemistry, University of Malaya, 50603 Kuala Lumpur, Malaysia; c Chemistry Department, Faculty of Science, King Abdulaziz University, PO Box 80203 Jeddah, Saudi Arabia

## Abstract

The pyrimidine ring of the title compound, C_25_H_30_N_2_O_3_, is approximately planar (r.m.s. deviation = 0.003 Å); the C atom at the 5-position deviates by 0.012 (3) Å from the mean plane and the C atom at the 6-position by 0.038 (3) Å. In the mol­ecule, the pyrimidine ring is oriented at 86.72 (9) and 59.75 (9)° with respect to the two benzene rings, and the two benzene rings are inclined to each other at 58.35 (9)°. In the crystal, the amino group is hydrogen-bond donor to the exocyclic O atom at the 4-position of an adjacent mol­ecule, the hydrogen bond generating an inversion dimer.

## Related literature

For the applications and synthesis of the title compound, see: El-Brollosy *et al.* (2009 ▶).
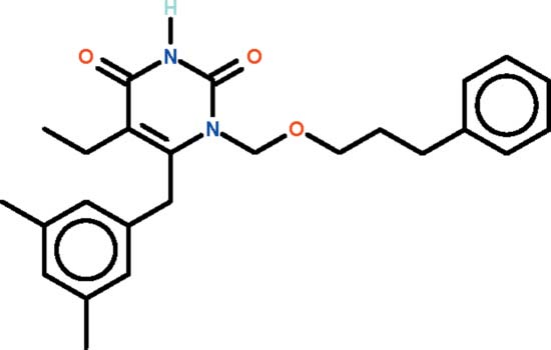


## Experimental

### 

#### Crystal data

C_25_H_30_N_2_O_3_
*M* *_r_* = 406.51
Triclinic, 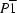

*a* = 4.8276 (4) Å
*b* = 14.9398 (10) Å
*c* = 15.4574 (12) Å
α = 76.949 (6)°
β = 89.296 (6)°
γ = 88.267 (6)°
*V* = 1085.53 (14) Å^3^
*Z* = 2
Mo *K*α radiation
μ = 0.08 mm^−1^
*T* = 100 K
0.25 × 0.20 × 0.05 mm

#### Data collection

Agilent SuperNova Dual diffractometer with an Atlas detector
Absorption correction: multi-scan (*CrysAlis PRO*; Agilent, 2010 ▶) *T*_min_ = 0.980, *T*_max_ = 0.996
7623 measured reflections
4974 independent reflections
3719 reflections with *I* > 2σ(*I*)
*R*_int_ = 0.039

#### Refinement

*R*[*F* ^2^ > 2σ(*F* ^2^)] = 0.056
*wR*(*F* ^2^) = 0.167
*S* = 1.04
4974 reflections
277 parameters
1 restraint
H atoms treated by a mixture of independent and constrained refinement
Δρ_max_ = 0.39 e Å^−3^
Δρ_min_ = −0.29 e Å^−3^

### 

Data collection: *CrysAlis PRO* (Agilent, 2010 ▶); cell refinement: *CrysAlis PRO*; data reduction: *CrysAlis PRO*; program(s) used to solve structure: *SHELXS97* (Sheldrick, 2008 ▶); program(s) used to refine structure: *SHELXL97* (Sheldrick, 2008 ▶); molecular graphics: *X-SEED* (Barbour, 2001 ▶); software used to prepare material for publication: *publCIF* (Westrip, 2010 ▶).

## Supplementary Material

Crystal structure: contains datablock(s) global, I. DOI: 10.1107/S1600536811055723/xu5425sup1.cif

Structure factors: contains datablock(s) I. DOI: 10.1107/S1600536811055723/xu5425Isup2.hkl

Supplementary material file. DOI: 10.1107/S1600536811055723/xu5425Isup3.cml

Additional supplementary materials:  crystallographic information; 3D view; checkCIF report

## Figures and Tables

**Table 1:** Hydrogen-bond geometry (Å, °)

*D*—H⋯*A*	*D*—H	H⋯*A*	*D*⋯*A*	*D*—H⋯*A*
N1—H1⋯O2^i^	0.89 (1)	1.94 (1)	2.826 (2)	178 (2)

Symmetry code: (i) 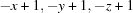.

**Fig. 1.**
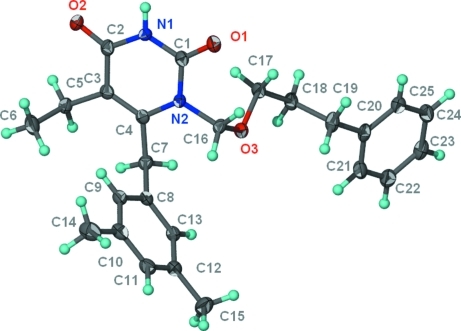
Thermal ellipsoid plot (Barbour, 2001) of C25H30N2O3 at the 70% probability level; hydrogen atoms are drawn as spheres of arbitrary radius.

**Fig. 2.**
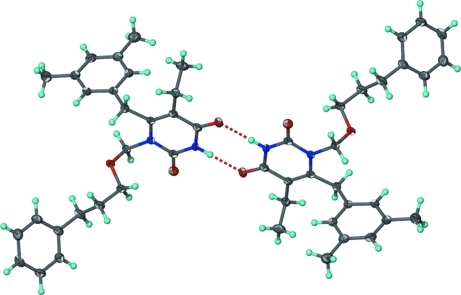
Dimeric hydrogen-bonded structure.

## supplementary crystallographic information

### Comment

The compound (Scheme I) was synthesized for an evaluation of its anti-viral activity against HIV-1 (El-Brollosy *et al.*, 2009). The pyrimidine ring is planar; the C atom at the 5-position deviates by 0.012 (3) Å from the mean plane and the C atom at the 6-position by 0.038 (3) Å (Fig. 1). The amino group is hydrogen-bond donor to the exocyclic O atom at the 4-position, the hydrogen bond generating a centrosymmetric dimer (Table 1, Fig. 1).

### Experimental

The compound was synthesized by using a reported method (El-Brollosy *et al.*, 2009), and was recrystallized from ethanol.

### Refinement

Carbon-bound H-atoms were placed in calculated positions [C–H 0.95 to 0.99 Å, *U*_iso_(H) 1.2 to 1.5*U*_eq_(C)] and were included in the refinement in the riding model approximation.

The amino H-atom was located in a difference Fourier map, and was refined with a distance restraint of N–H 0.88±0.01 Å; its temperature factor was refined.

### Crystal data

**Table:**

C_25_H_30_N_2_O_3_	*Z* = 2
*M**_r_* = 406.51	*F*(000) = 436
Triclinic, *P*1	*D*_x_ = 1.244 Mg m^−^^3^
Hall symbol: -P 1	Mo *K*α radiation, λ = 0.71073 Å
*a* = 4.8276 (4) Å	Cell parameters from 2909 reflections
*b* = 14.9398 (10) Å	θ = 2.7–27.5°
*c* = 15.4574 (12) Å	µ = 0.08 mm^−^^1^
α = 76.949 (6)°	*T* = 100 K
β = 89.296 (6)°	Prism, colorless
γ = 88.267 (6)°	0.25 × 0.20 × 0.05 mm
*V* = 1085.53 (14) Å^3^	

### Data collection

**Table:**

Agilent SuperNova Dual diffractometer with an Atlas detector	4974 independent reflections
Radiation source: SuperNova (Mo) X-ray Source	3719 reflections with *I* > 2σ(*I*)
Mirror	*R*_int_ = 0.039
Detector resolution: 10.4041 pixels mm^-1^	θ_max_ = 27.6°, θ_min_ = 2.7°
ω scan	*h* = −6→4
Absorption correction: multi-scan (CrysAlis PRO; Agilent, 2010)	*k* = −19→19
*T*_min_ = 0.980, *T*_max_ = 0.996	*l* = −20→18
7623 measured reflections	

### Refinement

**Table:**

Refinement on *F*^2^	Primary atom site location: structure-invariant direct methods
Least-squares matrix: full	Secondary atom site location: difference Fourier map
*R*[*F*^2^ > 2σ(*F*^2^)] = 0.056	Hydrogen site location: inferred from neighbouring sites
*wR*(*F*^2^) = 0.167	H atoms treated by a mixture of independent and constrained refinement
*S* = 1.04	*w* = 1/[σ^2^(*F*_o_^2^) + (0.0802*P*)^2^ + 0.2053*P*] where *P* = (*F*_o_^2^ + 2*F*_c_^2^)/3
4974 reflections	(Δ/σ)_max_ = 0.001
277 parameters	Δρ_max_ = 0.39 e Å^−^^3^
1 restraint	Δρ_min_ = −0.29 e Å^−^^3^

### Fractional atomic coordinates and isotropic or equivalent isotropic displacement parameters (Å^2^)

**Table:**

	*x*	*y*	*z*	*U*_iso_*/*U*_eq_	
O1	0.7285 (3)	0.74030 (9)	0.40923 (8)	0.0227 (3)	
O2	0.2592 (3)	0.47945 (8)	0.42401 (8)	0.0216 (3)	
O3	0.2541 (3)	0.89685 (8)	0.27377 (8)	0.0188 (3)	
N1	0.4879 (3)	0.61150 (10)	0.41488 (9)	0.0167 (3)	
H1	0.572 (4)	0.5837 (12)	0.4647 (8)	0.014 (5)*	
N2	0.4166 (3)	0.73931 (10)	0.29812 (9)	0.0151 (3)	
C1	0.5563 (4)	0.69986 (12)	0.37641 (11)	0.0169 (4)	
C2	0.2987 (4)	0.55921 (12)	0.38339 (11)	0.0174 (4)	
C3	0.1581 (4)	0.60349 (12)	0.30160 (11)	0.0160 (4)	
C4	0.2187 (4)	0.69150 (12)	0.26177 (11)	0.0156 (4)	
C5	−0.0516 (4)	0.54575 (12)	0.26870 (12)	0.0186 (4)	
H5A	−0.1999	0.5869	0.2367	0.022*	
H5B	−0.1376	0.5044	0.3205	0.022*	
C6	0.0717 (4)	0.48772 (14)	0.20714 (13)	0.0276 (5)	
H6A	−0.0749	0.4526	0.1881	0.041*	
H6B	0.2147	0.4453	0.2389	0.041*	
H6C	0.1542	0.5281	0.1550	0.041*	
C7	0.0796 (4)	0.74447 (12)	0.17790 (11)	0.0174 (4)	
H7A	0.0081	0.8038	0.1887	0.021*	
H7B	−0.0822	0.7096	0.1667	0.021*	
C8	0.2569 (4)	0.76452 (12)	0.09395 (11)	0.0165 (4)	
C9	0.4438 (4)	0.69892 (12)	0.07253 (12)	0.0184 (4)	
H9	0.4670	0.6407	0.1124	0.022*	
C10	0.5962 (4)	0.71790 (13)	−0.00650 (12)	0.0208 (4)	
C11	0.5603 (4)	0.80409 (13)	−0.06396 (12)	0.0217 (4)	
H11	0.6650	0.8178	−0.1175	0.026*	
C12	0.3753 (4)	0.87043 (13)	−0.04483 (12)	0.0230 (4)	
C13	0.2259 (4)	0.84895 (12)	0.03477 (12)	0.0195 (4)	
H13	0.0991	0.8936	0.0488	0.023*	
C14	0.7919 (4)	0.64610 (15)	−0.03046 (13)	0.0297 (5)	
H14A	0.9592	0.6757	−0.0584	0.045*	
H14B	0.7005	0.6158	−0.0719	0.045*	
H14C	0.8429	0.6003	0.0234	0.045*	
C15	0.3370 (5)	0.96336 (14)	−0.10683 (13)	0.0317 (5)	
H15A	0.3628	1.0115	−0.0741	0.048*	
H15B	0.1497	0.9693	−0.1316	0.048*	
H15C	0.4735	0.9697	−0.1551	0.048*	
C16	0.4702 (4)	0.83684 (11)	0.25921 (11)	0.0169 (4)	
H16A	0.6428	0.8536	0.2849	0.020*	
H16B	0.5000	0.8449	0.1945	0.020*	
C17	0.2175 (4)	0.90085 (12)	0.36571 (11)	0.0197 (4)	
H17A	0.3996	0.9041	0.3934	0.024*	
H17B	0.1242	0.8452	0.3989	0.024*	
C18	0.0417 (4)	0.98597 (12)	0.36809 (12)	0.0202 (4)	
H18A	−0.0194	0.9832	0.4300	0.024*	
H18B	−0.1260	0.9863	0.3318	0.024*	
C19	0.1936 (4)	1.07554 (12)	0.33360 (13)	0.0230 (4)	
H19A	0.3619	1.0752	0.3697	0.028*	
H19B	0.2536	1.0787	0.2715	0.028*	
C20	0.0166 (4)	1.15986 (12)	0.33684 (12)	0.0192 (4)	
C21	−0.1760 (4)	1.19492 (13)	0.27088 (12)	0.0226 (4)	
H21	−0.1878	1.1679	0.2210	0.027*	
C22	−0.3515 (4)	1.26880 (13)	0.27654 (13)	0.0248 (4)	
H22	−0.4838	1.2912	0.2312	0.030*	
C23	−0.3344 (4)	1.31006 (13)	0.34816 (13)	0.0240 (4)	
H23	−0.4567	1.3598	0.3528	0.029*	
C24	−0.1361 (4)	1.27772 (13)	0.41292 (12)	0.0232 (4)	
H24	−0.1187	1.3067	0.4612	0.028*	
C25	0.0366 (4)	1.20321 (12)	0.40744 (12)	0.0207 (4)	
H25	0.1703	1.1814	0.4524	0.025*	

### Atomic displacement parameters (Å^2^)

**Table:**

	*U*^11^	*U*^22^	*U*^33^	*U*^12^	*U*^13^	*U*^23^
O1	0.0282 (7)	0.0184 (7)	0.0228 (7)	−0.0030 (6)	−0.0061 (6)	−0.0063 (5)
O2	0.0311 (7)	0.0122 (6)	0.0209 (6)	−0.0008 (5)	−0.0045 (6)	−0.0022 (5)
O3	0.0256 (7)	0.0144 (6)	0.0171 (6)	0.0028 (5)	0.0006 (5)	−0.0060 (5)
N1	0.0237 (8)	0.0121 (7)	0.0137 (7)	0.0013 (6)	−0.0042 (6)	−0.0020 (6)
N2	0.0192 (7)	0.0118 (7)	0.0145 (7)	−0.0008 (6)	0.0002 (6)	−0.0036 (6)
C1	0.0189 (9)	0.0156 (9)	0.0175 (8)	0.0010 (7)	0.0010 (7)	−0.0067 (7)
C2	0.0201 (9)	0.0144 (8)	0.0190 (9)	0.0007 (7)	0.0032 (7)	−0.0067 (7)
C3	0.0174 (9)	0.0157 (9)	0.0160 (8)	0.0021 (7)	0.0010 (7)	−0.0065 (7)
C4	0.0183 (8)	0.0146 (9)	0.0147 (8)	0.0020 (7)	0.0019 (7)	−0.0057 (7)
C5	0.0208 (9)	0.0144 (9)	0.0205 (9)	−0.0008 (7)	−0.0019 (7)	−0.0039 (7)
C6	0.0350 (11)	0.0242 (10)	0.0277 (10)	−0.0064 (9)	0.0021 (9)	−0.0138 (8)
C7	0.0188 (9)	0.0152 (9)	0.0177 (8)	0.0009 (7)	0.0003 (7)	−0.0031 (7)
C8	0.0196 (9)	0.0159 (9)	0.0150 (8)	−0.0018 (7)	−0.0023 (7)	−0.0053 (7)
C9	0.0216 (9)	0.0168 (9)	0.0175 (8)	0.0004 (7)	−0.0040 (7)	−0.0053 (7)
C10	0.0206 (9)	0.0242 (10)	0.0195 (9)	0.0012 (8)	−0.0029 (7)	−0.0088 (8)
C11	0.0242 (10)	0.0257 (10)	0.0168 (9)	−0.0047 (8)	0.0026 (7)	−0.0076 (8)
C12	0.0336 (11)	0.0190 (9)	0.0170 (9)	−0.0042 (8)	−0.0011 (8)	−0.0047 (7)
C13	0.0263 (10)	0.0155 (9)	0.0177 (8)	0.0017 (7)	−0.0023 (7)	−0.0062 (7)
C14	0.0300 (11)	0.0360 (12)	0.0249 (10)	0.0083 (9)	0.0005 (8)	−0.0117 (9)
C15	0.0512 (14)	0.0206 (10)	0.0223 (10)	−0.0020 (9)	0.0085 (9)	−0.0033 (8)
C16	0.0214 (9)	0.0117 (8)	0.0174 (8)	0.0006 (7)	0.0025 (7)	−0.0032 (7)
C17	0.0275 (10)	0.0169 (9)	0.0162 (8)	−0.0011 (8)	0.0036 (7)	−0.0067 (7)
C18	0.0242 (9)	0.0174 (9)	0.0212 (9)	−0.0025 (7)	0.0046 (7)	−0.0087 (7)
C19	0.0246 (10)	0.0162 (9)	0.0304 (10)	−0.0007 (8)	0.0049 (8)	−0.0097 (8)
C20	0.0205 (9)	0.0143 (9)	0.0237 (9)	−0.0055 (7)	0.0068 (7)	−0.0058 (7)
C21	0.0276 (10)	0.0193 (9)	0.0233 (9)	−0.0048 (8)	0.0020 (8)	−0.0092 (8)
C22	0.0259 (10)	0.0195 (10)	0.0282 (10)	−0.0029 (8)	−0.0055 (8)	−0.0032 (8)
C23	0.0249 (10)	0.0150 (9)	0.0320 (10)	−0.0001 (8)	0.0035 (8)	−0.0055 (8)
C24	0.0303 (10)	0.0195 (9)	0.0219 (9)	−0.0023 (8)	0.0043 (8)	−0.0094 (8)
C25	0.0231 (9)	0.0175 (9)	0.0215 (9)	−0.0027 (7)	0.0002 (7)	−0.0041 (7)

### Geometric parameters (Å, °)

**Table:**

O1—C1	1.222 (2)	C12—C13	1.397 (3)
O2—C2	1.234 (2)	C12—C15	1.506 (3)
O3—C16	1.404 (2)	C13—H13	0.9500
O3—C17	1.444 (2)	C14—H14A	0.9800
N1—C1	1.368 (2)	C14—H14B	0.9800
N1—C2	1.381 (2)	C14—H14C	0.9800
N1—H1	0.885 (9)	C15—H15A	0.9800
N2—C1	1.393 (2)	C15—H15B	0.9800
N2—C4	1.405 (2)	C15—H15C	0.9800
N2—C16	1.475 (2)	C16—H16A	0.9900
C2—C3	1.454 (2)	C16—H16B	0.9900
C3—C4	1.359 (2)	C17—C18	1.514 (2)
C3—C5	1.514 (2)	C17—H17A	0.9900
C4—C7	1.511 (2)	C17—H17B	0.9900
C5—C6	1.529 (3)	C18—C19	1.530 (3)
C5—H5A	0.9900	C18—H18A	0.9900
C5—H5B	0.9900	C18—H18B	0.9900
C6—H6A	0.9800	C19—C20	1.511 (2)
C6—H6B	0.9800	C19—H19A	0.9900
C6—H6C	0.9800	C19—H19B	0.9900
C7—C8	1.523 (2)	C20—C21	1.390 (3)
C7—H7A	0.9900	C20—C25	1.395 (3)
C7—H7B	0.9900	C21—C22	1.389 (3)
C8—C13	1.386 (2)	C21—H21	0.9500
C8—C9	1.404 (2)	C22—C23	1.388 (3)
C9—C10	1.395 (2)	C22—H22	0.9500
C9—H9	0.9500	C23—C24	1.389 (3)
C10—C11	1.397 (3)	C23—H23	0.9500
C10—C14	1.513 (3)	C24—C25	1.388 (3)
C11—C12	1.393 (3)	C24—H24	0.9500
C11—H11	0.9500	C25—H25	0.9500
			
C16—O3—C17	113.95 (13)	C10—C14—H14A	109.5
C1—N1—C2	126.47 (14)	C10—C14—H14B	109.5
C1—N1—H1	118.3 (13)	H14A—C14—H14B	109.5
C2—N1—H1	115.2 (13)	C10—C14—H14C	109.5
C1—N2—C4	122.09 (14)	H14A—C14—H14C	109.5
C1—N2—C16	116.28 (14)	H14B—C14—H14C	109.5
C4—N2—C16	121.44 (14)	C12—C15—H15A	109.5
O1—C1—N1	121.59 (16)	C12—C15—H15B	109.5
O1—C1—N2	123.13 (16)	H15A—C15—H15B	109.5
N1—C1—N2	115.28 (15)	C12—C15—H15C	109.5
O2—C2—N1	119.97 (15)	H15A—C15—H15C	109.5
O2—C2—C3	123.88 (16)	H15B—C15—H15C	109.5
N1—C2—C3	116.15 (15)	O3—C16—N2	113.40 (14)
C4—C3—C2	119.14 (16)	O3—C16—H16A	108.9
C4—C3—C5	125.36 (15)	N2—C16—H16A	108.9
C2—C3—C5	115.50 (15)	O3—C16—H16B	108.9
C3—C4—N2	120.86 (15)	N2—C16—H16B	108.9
C3—C4—C7	123.32 (16)	H16A—C16—H16B	107.7
N2—C4—C7	115.82 (14)	O3—C17—C18	107.67 (14)
C3—C5—C6	113.79 (15)	O3—C17—H17A	110.2
C3—C5—H5A	108.8	C18—C17—H17A	110.2
C6—C5—H5A	108.8	O3—C17—H17B	110.2
C3—C5—H5B	108.8	C18—C17—H17B	110.2
C6—C5—H5B	108.8	H17A—C17—H17B	108.5
H5A—C5—H5B	107.7	C17—C18—C19	113.32 (15)
C5—C6—H6A	109.5	C17—C18—H18A	108.9
C5—C6—H6B	109.5	C19—C18—H18A	108.9
H6A—C6—H6B	109.5	C17—C18—H18B	108.9
C5—C6—H6C	109.5	C19—C18—H18B	108.9
H6A—C6—H6C	109.5	H18A—C18—H18B	107.7
H6B—C6—H6C	109.5	C20—C19—C18	112.78 (15)
C4—C7—C8	116.78 (14)	C20—C19—H19A	109.0
C4—C7—H7A	108.1	C18—C19—H19A	109.0
C8—C7—H7A	108.1	C20—C19—H19B	109.0
C4—C7—H7B	108.1	C18—C19—H19B	109.0
C8—C7—H7B	108.1	H19A—C19—H19B	107.8
H7A—C7—H7B	107.3	C21—C20—C25	118.12 (17)
C13—C8—C9	118.59 (16)	C21—C20—C19	120.98 (16)
C13—C8—C7	119.19 (16)	C25—C20—C19	120.87 (17)
C9—C8—C7	122.17 (15)	C22—C21—C20	121.14 (17)
C10—C9—C8	120.99 (17)	C22—C21—H21	119.4
C10—C9—H9	119.5	C20—C21—H21	119.4
C8—C9—H9	119.5	C23—C22—C21	120.25 (18)
C11—C10—C9	118.59 (17)	C23—C22—H22	119.9
C11—C10—C14	120.58 (17)	C21—C22—H22	119.9
C9—C10—C14	120.82 (17)	C22—C23—C24	119.15 (17)
C12—C11—C10	121.78 (17)	C22—C23—H23	120.4
C12—C11—H11	119.1	C24—C23—H23	120.4
C10—C11—H11	119.1	C25—C24—C23	120.34 (17)
C11—C12—C13	118.06 (17)	C25—C24—H24	119.8
C11—C12—C15	121.73 (17)	C23—C24—H24	119.8
C13—C12—C15	120.21 (17)	C24—C25—C20	120.95 (17)
C8—C13—C12	121.99 (17)	C24—C25—H25	119.5
C8—C13—H13	119.0	C20—C25—H25	119.5
C12—C13—H13	119.0		
			
C2—N1—C1—O1	−179.48 (16)	C7—C8—C9—C10	177.55 (16)
C2—N1—C1—N2	0.4 (2)	C8—C9—C10—C11	0.4 (3)
C4—N2—C1—O1	−179.83 (16)	C8—C9—C10—C14	−178.39 (17)
C16—N2—C1—O1	−4.8 (2)	C9—C10—C11—C12	−0.8 (3)
C4—N2—C1—N1	0.3 (2)	C14—C10—C11—C12	178.03 (17)
C16—N2—C1—N1	175.32 (14)	C10—C11—C12—C13	0.6 (3)
C1—N1—C2—O2	178.90 (16)	C10—C11—C12—C15	179.97 (19)
C1—N1—C2—C3	−0.5 (3)	C9—C8—C13—C12	−0.3 (3)
O2—C2—C3—C4	−179.46 (17)	C7—C8—C13—C12	−177.79 (16)
N1—C2—C3—C4	−0.1 (2)	C11—C12—C13—C8	−0.1 (3)
O2—C2—C3—C5	1.5 (2)	C15—C12—C13—C8	−179.45 (18)
N1—C2—C3—C5	−179.14 (15)	C17—O3—C16—N2	66.98 (18)
C2—C3—C4—N2	0.7 (2)	C1—N2—C16—O3	−103.56 (17)
C5—C3—C4—N2	179.69 (15)	C4—N2—C16—O3	71.52 (19)
C2—C3—C4—C7	−178.51 (15)	C16—O3—C17—C18	163.75 (14)
C5—C3—C4—C7	0.5 (3)	O3—C17—C18—C19	−70.60 (19)
C1—N2—C4—C3	−0.9 (2)	C17—C18—C19—C20	−179.67 (15)
C16—N2—C4—C3	−175.63 (15)	C18—C19—C20—C21	−81.0 (2)
C1—N2—C4—C7	178.43 (14)	C18—C19—C20—C25	97.2 (2)
C16—N2—C4—C7	3.6 (2)	C25—C20—C21—C22	−2.5 (3)
C4—C3—C5—C6	91.8 (2)	C19—C20—C21—C22	175.80 (17)
C2—C3—C5—C6	−89.18 (19)	C20—C21—C22—C23	1.0 (3)
C3—C4—C7—C8	−110.06 (19)	C21—C22—C23—C24	1.3 (3)
N2—C4—C7—C8	70.7 (2)	C22—C23—C24—C25	−2.0 (3)
C4—C7—C8—C13	−141.21 (17)	C23—C24—C25—C20	0.5 (3)
C4—C7—C8—C9	41.3 (2)	C21—C20—C25—C24	1.7 (3)
C13—C8—C9—C10	0.1 (3)	C19—C20—C25—C24	−176.55 (17)

### Hydrogen-bond geometry (Å, °)

**Table:**

*D*—H···*A*	*D*—H	H···*A*	*D*···*A*	*D*—H···*A*
N1—H1···O2^i^	0.89 (1)	1.94 (1)	2.826 (2)	178 (2)

Symmetry codes: (i) −*x*+1, −*y*+1, −*z*+1.
